# L-Dopa/Carbidopa intestinal gel infusion in advanced Parkinson’s disease: real-life mobility insights from wearable sensors

**DOI:** 10.3389/fneur.2026.1812950

**Published:** 2026-06-15

**Authors:** Alessandro Zampogna, Luigi Borzì, Domiziana Rinaldi, Gabriele Imbalzano, Martina Patera, Marco Falletti, Carlo Alberto Artusi, Edoardo Bianchini, Leonardo Lopiano, Gabriella Olmo, Antonio Suppa

**Affiliations:** 1 Department of Human Neurosciences, Sapienza University of Rome, Rome, Italy; 2 IRCCS Neuromed Institute, Pozzilli, Italy; 3 Department of Control and Computer Engineering, Politecnico di Torino, Turin, Italy; 4 Department of Neuroscience, Mental Health and Sense Organs (NESMOS), Sapienza University of Rome, Rome, Italy; 5 Department of Neuroscience “Rita Levi Montalcini”, University of Turin, Torino, Italy; 6 Department of Neurosciences, Biomedicine, and Movement, University of Verona, Verona, Italy; 7 Fondazione Policlinico Universitario Campus Bio-Medico, Rome, Italy; 8 Université Grenoble Alpes, CNRS, Grenoble INP, LIG Sangria, Grenoble, France; 9 Neurology 2 Unit, A.O.U Città della Salute e della Scienza di Torino, Torino, Italy

**Keywords:** fluctuations, LCIG, motor complications, Parkinson’s disease, wearable sensors

## Abstract

**Rationale/objectives:**

In advanced Parkinson’s disease (APD), the intermittent dopaminergic delivery of oral L-Dopa contributes to the occurrence of fluctuations and dyskinesia. Continuous dopaminergic delivery through intrajejunal L-Dopa/Carbidopa intestinal gel (LCIG) is an established therapeutic strategy to manage these motor complications, but its impact on real-life motor performances has never been characterised objectively. This cross-sectional pilot study used wearable sensors to quantitatively evaluate the impact of LCIG on patients’ motor performance in real-world settings.

**Material/methods:**

Forty-three APD patients, including 15 treated with LCIG infusion (APD_LCIG_) and 28 on the best oral dopaminergic therapy (APD_L-Dopa_), were evaluated using standardized clinical scales and sensor-based metrics. A validated waist-worn inertial wearable, positioned on the left side, continuously monitored spatiotemporal gait parameters (step length, stride speed, stride fluidity, and cadence) along with freezing of gait (FOG) occurrence, and time spent with dyskinesia. To quantify intra-day variability in motor performance associated with motor fluctuations, the coefficient of variation of all spatiotemporal gait parameters was calculated. Finally, clinical-behavioural correlations were analysed to examine possible relationships between gait features and clinical outcomes.

**Results:**

Despite comparable clinical and demographic profiles, APD_LCIG_ showed less severe motor fluctuations than APD_L-Dopa_ and exhibited lower intra-day variability in key spatiotemporal gait parameters (step length, stride speed, and stride fluidity). Dyskinesia duration was similar in APD_LCIG_ and APD_L-Dopa_. Also, mean absolute values of gait parameters and FOG duration did not differ between groups. Stride fluidity, step length, and stride speed were moderately and inversely associated with age and disease severity.

**Discussion/conclusion:**

LCIG provides a more stable gait pattern than optimized oral dopaminergic therapy in appropriately selected APD patients, as captured by wearable sensors in real-world conditions. This likely reflects more consistent motor control throughout the day due to continuous dopaminergic delivery. By detecting fluctuation-related motor impairment through variability metrics, wearable sensor technology may offer a valuable tool to enhance the clinical management of LCIG, supporting patient selection, medication titration, and the longitudinal monitoring of treatment efficacy and safety.

## 1 Introduction

The advanced stage of Parkinson’s disease (APD) is characterized by fluctuating symptoms, including motor fluctuations and dyskinesias (i.e., drug-induced involuntary movements), as motor control becomes increasingly reliant on the intermittent intake of oral medications (1). L-Dopa/Carbidopa intestinal gel (LCIG) infusion is a leading therapeutic strategy for managing these motor complications in APD (2). This approach involves the continuous delivery of tailored doses of L-Dopa/Carbidopa directly to the jejunum via an electronic pump and a thin tube inserted through a percutaneous endoscopic gastrostomy with a jejunal extension (PEG-J) (2). Multiple clinical studies have demonstrated that LCIG effectively reduces motor fluctuations and dyskinesias in APD patients while increasing ON time and reducing OFF time (3–6). However, the available evidence mostly derives from regulatory trials and subsequent observational studies that relied on qualitative assessment tools, such as clinical scales and patient diaries (3, 7–9). These instruments are prone to recall bias, suffer from inter- and intra-rater variability, and lack the sensitivity to accurately capture brief or fluctuating motor abnormalities, like fluctuations and dyskinesias (10, 11). Moreover, the in-hospital clinical examination provides only a single snapshot of the patient’s condition and cannot capture the full movie of motor performance variability throughout the day. This is particularly relevant in the context of APD, since symptoms fluctuation is one of the primary indications for device-aided therapies. Long-term, quantitative monitoring of motor symptoms through instrumental methods would substantially improve the characterization of intra-day variability in real-world conditions, thereby strengthening all phases of LCIG referral and management.

Recent advances in wearable technology have enabled long-term, real-world monitoring of PD using small inertial measurement units (IMUs) (12, 13). Combined with specialized algorithms, these sensors provide sensitive and objective detection of motor disorders in both controlled and ecological environments (14–17). IMUs have proven effective in quantifying motor disturbances, offering valuable insight into symptom variability and progression (18–20), with high patient adherence and acceptance (21, 22). Several studies have suggested the potential of wearable data to support patient selection for device-aided therapies and to refine treatment strategies (23–29). Among the motor functions measurable with these devices, gait has emerged as a sensitive marker of patients’ therapeutic state, showing quantifiable fluctuations in parallel with medication effects throughout the day (30, 31). Accordingly, evaluating the variability of spatiotemporal gait parameters in APD could provide a robust tool to quantify fluctuations and assess the stability of motor performance in real-life conditions (13).

Nevertheless, to date, only a few studies have applied wearable sensors to support clinical decision-making in APD patients treated with LCIG (APD_LCIG_). Kilinçalp and colleagues (28) investigated the prognostic value of sensor-based measures for identifying suitable candidates for LCIG, showing that patients with clearly identifiable OFF periods at baseline were more likely to benefit from the therapy. Additionally, Imbalzano and colleagues (32) used wearable sensors in a controlled hospital setting to quantify axial motor symptoms in APD_LCIG_, demonstrating a positive effect of LCIG on spatiotemporal gait parameters. However, to the best of our knowledge, no studies have assessed gait in APD patients with and without LCIG by using long-term sensor-based monitoring to capture intra-day motor variability in real-world settings.

In this cross-sectional pilot study, we performed and compared long-term gait monitoring in APD_LCIG_ and those receiving the best oral dopaminergic therapy (APD_L-Dopa_), using a validated wearable sensor in real-world settings. The primary aim was to objectively determine whether LCIG is associated with more stable motor control than oral dopaminergic therapy, as reflected by spatiotemporal gait parameters. We hypothesised that APD_LCIG_ would show lower variability in gait metrics than APD_L-Dopa_, reflecting the stabilizing effect of continuous dopaminergic delivery. We also took the opportunity to briefly discuss the potential practical applications of wearable sensors in the clinical management of LCIG therapy.

## 2 Materials and methods

This multicenter, cross-sectional pilot study was conducted following the guidelines outlined in the “Strengthening the Reporting of Observational Studies in Epidemiology” (STROBE) statement. The study protocol was approved by the Institutional Review Board of Sapienza University of Rome, Italy (Prot. 0372/2022). All participants provided written informed consent, in compliance with the principles of the Declaration of Helsinki.

### 2.1 Subjects and clinical evaluation

We consecutively screened patients with APD undergoing neurological evaluation at the movement disorder outpatient clinics of Sapienza University of Rome and the University of Turin (Italy) between January 2023 and September 2025 for potential enrollment, according to the following inclusion criteria: diagnosis of idiopathic PD based on current consensus criteria and follow-up clinical evaluations (33), advanced disease stage according to the “5–2-1” criteria (including ≥ 5 doses of oral levodopa per day and/or ≥ 2 h of “off” time per day, and/or ≥ 1 h of troublesome dyskinesia per day) (34) or ongoing treatment with LCIG as a device-aided therapy for PD for at least 3 months; ability to walk independently (Hoehn and Yahr scale – H&Y ≤ 4). We excluded patients with a diagnosis of possible or probable atypical parkinsonism; device-aided therapies other than LCIG infusion (e.g., subcutaneous apomorphine or L-Dopa infusion; deep brain stimulation); previous MRI-guided focused ultrasound or radiofrequency lesioning surgery; inability to walk independently; severe cognitive impairment (Montreal cognitive assessment – MoCA < 18); comorbidities (e.g., neurological conditions other than PD, orthopedic and/or rheumatologic issues, or determining using chronic medications potentially affecting gait). We used standardized protocols to calculate the L-Dopa equivalent daily doses (LEDDs) for each patient (35). No participants were receiving other neuropsychiatric medications possibly affecting gait during the study period.

Before initiating the home-based gait monitoring with wearable sensors, all participants were assessed in the outpatient clinic by a movement disorder specialist. The evaluation included a structured battery of validated clinical scales: H&Y; Movement Disorder Society–Unified Parkinson’s Disease Rating Scale (MDS-UPDRS) part III; Unified Dyskinesia Rating Scale (UDysRS) parts III–IV; the Wearing-Off Questionnaire-19 (WOQ-19); MoCA; frontal assessment battery (FAB); Beck depression inventory II (BDI-II); and Beck anxiety inventory (BAI). To capture prevalent clinical conditions at home, all clinical assessments were performed in the ON state (i.e., 1 h after the L-Dopa dose in APD_L-Dopa_ and at least 1 h after pump activation in the APD_LCIG_, once the infusion had achieved a stable therapeutic level).

### 2.2 Wearable sensor and long-term monitoring

In addition to clinical assessments using standardized scales, patients underwent long-term gait monitoring with a validated wearable sensor (STAT-ON™, Sense4Care, Barcelona, Spain). STAT-ON™ is an inertial medical device developed and licensed for the continuous monitoring of motor symptoms in patients with PD during daily activities (36). The device is compact, measuring 9 × 6.3 × 2.1 cm and weighing 86 g, and features two ultra-low triaxial nano-accelerometers, two microcontrollers, and a Bluetooth low-energy communication system (37). Its accelerometer operates within a ± 6 g range, offers a resolution of 12 bits, and has a sampling rate of 50 Hz, with a power consumption of 12 μA and a battery life of up to 7 days under normal operating conditions. STAT-ON™ captures spatiotemporal gait parameters, such as step length, stride speed, stride fluidity and cadence, while utilizing advanced machine learning algorithms to automatically detect dyskinesia and freezing of gait (FOG). Data are stored in internal memory and can be downloaded to mobile devices through a specific application. The device has been certified as a Class IIa Medical Device and has successfully passed comprehensive testing for safe use in home environments (37).

In accordance with previously-validated protocols (37, 38), patients were instructed to wear the sensor on the left side of the waist using an elastic belt for a minimum of 4 days, ensuring at least 8 h of wear each day during waking hours. Caregiver support was encouraged to facilitate proper device use when necessary. The device operated automatically for its entire battery life without the need for manual activation or deactivation. Detailed instructions were provided for the proper positioning and usage of the wearable sensor. The device was positioned such that the x, y, and z axes of the embedded sensors corresponded to the anterior, vertical (upward), and lateral (left) directions, respectively. Medical staff preconfigured the wearable sensor with the patient’s clinical data (e.g., H&Y stage, age, and lower limb length measured from the left anterior-superior iliac spine to the ground). After completing the monitoring period and returning the wearable device, each patient attended a second clinical visit to assess compliance with sensor use and to retrieve the recorded data for later offline analysis.

### 2.3 Sensor-embedded algorithms and measures

Acceleration data were processed directly by the device using sensor-embedded algorithms. Feature extraction, gait detection, computation of spatiotemporal gait parameters, and motor symptom classification (including dyskinesia and FOG) were automatically performed onboard using previously validated machine learning models. The implemented algorithms, based on support vector machine (SVM) classifiers and both temporal and frequency-domain features, have been described and validated in prior studies (39–42). The device outputs precomputed gait metrics, including number of steps, step length, stride speed, cadence, and stride fluidity, as well as summary measures of dyskinesia and FOG. These outputs were exported and used for subsequent analysis without additional signal-level processing. A brief description of the underlying processing approach is provided below for completeness, while full methodological details are available in the referenced validation studies (39–42). Acceleration data is divided into 3.2-s segments with a 50% overlap, and gait detection is carried out using a SVM classifier with a radial basis function kernel. The SVM model adopts two input features based on energy levels within the frequency bands of [0.1, 3] Hz and [0.1, 10] Hz. Strides are identified by detecting minima in the forward acceleration signal, excluding the first and last strides to ensure greater consistency. Key gait parameters, including number of steps, step length (estimated through the inverse pendulum model) (39), stride speed (forward velocity per stride), cadence (steps per minute), and stride fluidity (computed as the energy content in the 0.1–10 Hz frequency band) (41), are subsequently extracted and provided as average values over one-minute time frames. Dyskinesia detection is triggered when energy levels in the 1–4 Hz frequency band exceed a predetermined threshold while remaining below a threshold in the 0–20 Hz range for a minimum of 6 s, reducing false positives from voluntary movements (40). For FOG detection, the SVM model incorporates both temporal and spectral features (means, integrals, temporal and spectral kurtosis, autoregression coefficients) derived from three-axis accelerometer data (42). The system computes the time spent in dyskinesia every 10 min and records the number and duration of FOG episodes every minute. To optimize the technical reliability of kinematic data, analyses were limited to the three days following the clinical visit, excluding the first day to allow sensor familiarization and minimize behavioural adaptation effects. Moreover, one-minute epochs with an average walking-bout length ≤6 steps were excluded from the analysis of gait parameters. This approach allows to minimize estimation variability, thereby ensuring robust and comparable gait parameter quantification across subjects.

**Figure 1:**
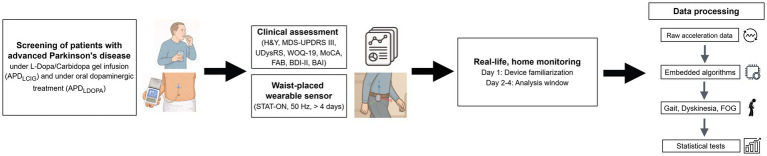
Study workflow. Patients with advanced Parkinson’s disease receiving either levodopa/carbidopa intestinal gel infusion (APD_LCIG_) or optimized oral dopaminergic therapy (APD_L-Dopa_) underwent a structured study protocol including clinical assessment and continuous home-based monitoring using a waist-worn wearable sensor. Following an initial familiarization day to ensure proper device use, real-world data were collected over four consecutive days during waking hours. The wearable device recorded triaxial acceleration signals, which were processed using validated embedded algorithms to derive spatiotemporal gait parameters, as well as motor complication metrics such as dyskinesia and freezing of gait. Extracted features were aggregated over the monitoring period and subsequently analyzed using appropriate statistical methods to compare motor performance and variability between groups.

The overall study workflow, including patient selection, data collection, processing, and analysis, is displayed in Figure 1.

### 2.4 Statistical analysis

Due to the exploratory nature of the study, no formal *a priori* sample size calculation was conducted. As each participant contributed to a single aggregated measure and no temporal changes were analysed, the study was treated as cross-sectional. An iterative matching procedure was applied, selecting subsets of APD_LCIG_ and APD_L-dopa_ to optimize baseline comparability while preserving the largest feasible sample size. Matching was performed on age, disease duration, and H&Y stage to ensure clinical balance between groups. More in detail, multiple combinations of patients were iteratively evaluated, and subsets were retained when they minimized between-group differences across these variables while maintaining adequate group sizes. The final matched sample was selected based on the best achievable balance across matching variables, as assessed by descriptive comparisons, acknowledging the exploratory nature of this approach.

Descriptive statistics were applied to summarize the demographic and clinical characteristics of APD patients, with continuous variables reported as mean ± standard deviation. The Shapiro–Wilk test was applied to assess normality of distribution for all continuous variables, revealing non-normal distributions for several key measures. Accordingly, the Mann–Whitney U-test was used to compare demographic and clinical features as well as sensor-based measures between patient subgroups (APD_L-Dopa_ vs. APD_LCIG_), with statistical significance defined as *p* < 0.05. Categorical variables, when present, were compared using the chi-square test.

To quantify motor fluctuations and assess the stability of patients’ motor performances in real-world settings, the coefficient of variation (CV) was calculated and compared for all gait spatiotemporal parameters, including step length, stride speed, cadence, and stride fluidity. The CV was computed as:


$$\mathrm{CV}=\frac{\mathrm{SD}}{\text{Mean}}$$

where *SD* denotes the standard deviation of each parameter and *Mean* represents its average value. For each participant, the CV was calculated using all measurements collected over the full 3-day monitoring period. Lastly, to investigate potential clinical–behavioural correlations between sensor-derived measures and clinical scores, Spearman’s correlation analysis was also performed. Adjustment for multiple testing was applied to the outcomes using the Benjamini–Hochberg correction (False Discovery Rate – FDR), and effect sizes were calculated using Cliff’s delta (*δ*) when appropriate.

Statistical analysis was performed using the SPSS package, Version 29.0 (IBM, Armonk, NY, USA). Only datasets meeting predefined quality and wear-time criteria were included, resulting in complete data for all analyzed participants. A thorough pre-analysis review confirmed the absence of missing values. For all statistical tests, the significance level was set at *α* < 0.05 (2-tailed).

## 3 Results

From the APD cohort screened during routine neurological evaluations, 79 patients underwent clinical evaluations and long-term gait monitoring with the wearable sensor at home. Of these, 21 were receiving LCIG infusion, and 58 were treated exclusively with oral dopaminergic therapy. After the matching procedure to improve baseline comparability between groups, the final analytical sample comprised a total of 43 subjects, including 15 APD_LCIG_ (9 men and 6 women) and 28 APD_L-Dopa_ (22 men and 6 women). All 43 enrolled participants completed the study protocol without deviations or technical issues. All participants adhered to the compliance monitoring requirements and completed the sensor-based gait monitoring as requested. All subjects showed optimal adherence to the wearable device, complying with recording instructions and ensuring at least 8 h of monitoring per day for a minimum of 4 days.

### 3.1 Clinical features

When comparing baseline demographic and clinical variables, the two subgroups of patients resulted well balanced in terms of demographic features, disease stage and severity, cognitive status, anxiety and mood symptoms, and LEDD (all factors that could otherwise influence the interpretation of spatiotemporal gait parameters). While the severity of dyskinesia, assessed through UDysRS parts III and IV, was comparable between groups, APD_LCIG_ reported lower WOQ-19 scores, indicating fewer self-reported symptoms fluctuations compared to APD_L-Dopa_. All the clinical and demographic variables included in the analysis are summarized in Table 1.

**Table 1:** Demographic and clinical features of patients with advanced Parkinson’s disease.

Variable*	APD_LCIG_ (*N =* 15)	APD_L-Dopa_ (*N =* 28)	*U-*statistic, *p*-value
Age	77.1 ± 18.6	67.0 ± 11.5	U = 146.0, *p* = 0.105
Disease duration	12.4 ± 5.7	10.0 ± 3.1	U = 161.0, *p* = 0.213
Age at onset	58.5 ± 10.3	56.2 ± 11.4	U = 194.5, *p* = 0.702
Hoehn and Yahr	2.4 ± 0.5	2.2 ± 0.5	U = 181.5, *p* = 0.406
MDS-UPDRS III	24.4 ± 13.5	23.8 ± 9.4	U = 174.0, *p* = 0.690
UDysRS III	3.6 ± 4.2	3.6 ± 4.6	U = 193.5, *p* = 0.676
UDysRS IV	2.8 ± 3.8	2.4 ± 2.8	U = 208.0, *p* = 0.968
WOQ-19	3.6 ± 2.7	6.4 ± 4.1	U = 122.5, ***p* = 0.026**
FOG-Q	6.6 ± 5.2	7.0 ± 6.1	U = 161.5, *p* = 0.831
MOCA	24.2 ± 3.8	25.1 ± 3.1	U = 177.0, *p* = 0.405
FAB	13.5 ± 1.6	14.7 ± 2.8	U = 137.0, *p* = 0.062
BDI-II	6.8 ± 5.1	8.4 ± 7.2	U = 127.0, *p* = 0.464
BAI	10.2 ± 13.3	10.5 ± 9.9	U = 26.5, *p* = 0.542
LEDDs	1206.6 ± 492.0	1066.3 ± 478.0	U = 165.0, *p* = 0.331

APD_LCIG_, patients with advanced Parkinson’s disease treated with L-Dopa/Carbidopa intestinal gel infusion; APD_L-Dopa_, patients with advanced Parkinson’s disease on best medical treatment with oral L-Dopa; BAI, Beck Anxiety Inventory; BDI-II, Beck Depression Inventory–II; FAB, Frontal Assessment Battery; FOG-Q, freezing of gait questionnaire; LEDDs, Levodopa Equivalent Daily Doses; MoCA, Montreal Cognitive Assessment; MDS-UPDRS, Movement Disorder Society–Unified Parkinson’s Disease Rating Scale; UDysRS, Unified Dyskinesia Rating Scale; WOQ-19, Wearing-Off Questionnaire–19. *All clinical assessments were performed in the ON state of therapy. Bold values indicate statistically significant results.

### 3.2 Sensor-derived measures

**Figure 2:**
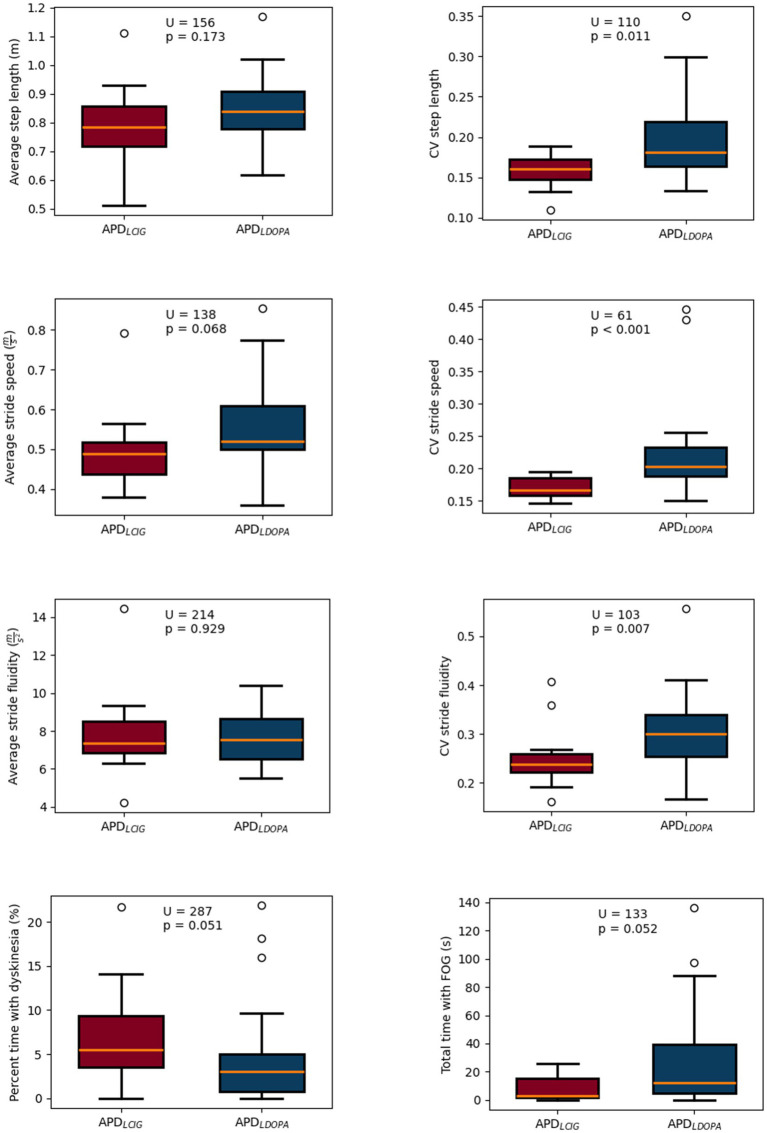
Main sensor-based measures. Wearable-derived spatiotemporal gait metrics in patients with Parkinson’s disease under L-Dopa/Carbidopa Gel Infusion (LCGI) and those under oral dopaminergic treatment (LDOPA). Boxplots illustrate group distributions of key spatiotemporal gait parameters derived from continuous home-based monitoring using a waist-worn wearable sensor, including total number of steps, cadence, step length, stride speed, and stride fluidity, along with their corresponding coefficients of variation (CV) as measures of gait variability. Central lines indicate medians, boxes represent interquartile ranges, and whiskers denote standard deviations.

Sensor-based analyses revealed some differences in mobility patterns of the two patients’ groups. More in detail, during the monitoring period, APD_LCIG_ took a similar number of steps and presented similar average values of step length, stride speed, cadence, and stride fluidity compared with APD_L-Dopa_ (all *p* > 0.05). Also, total time and percentage of time spent with dyskinesia, as well as time spent with FOG did not differ between groups (all p > 0.05). However, variability in spatiotemporal gait parameters was consistently reduced in APD_LCIG_, with significantly lower CV for stride speed (*p* < 0.001), step length (*p* = 0.011) and stride fluidity (*p* = 0.007). These differences in gait variability parameters remained significant after correction for multiple comparisons (FDR, *p* < 0.05) and were supported by moderate-to-large effect sizes, as indicated by Cliff’s delta (*δ* ranging from −0.48 to −0.71) (Table 2; Figure 2).

**Table 2:** Gait parameters of patients with advanced Parkinson’s disease.

Variable	APD_LCIG_ (*N =* 15)	APD_L-Dopa_ (*N =* 28)	*p*-value (adjusted)	Cliff’s δ
Total number of steps	32500.5 ± 18905.1	26994.1 ± 18420.3	U = 166, *p* = 0.268 (0.344)	−0.21
Average cadence	38.1 ± 3.2	38.8 ± 3.1	U = 175, *p* = 0.379 (0.426)	−0.17
CV cadence	0.11 ± 0.02	0.12 ± 0.02	U = 137, *p* = 0.065 (0.122)	−0.35
Average step length	0.79 ± 0.14	0.84 ± 0.12	U = 156, *p* = 0.173 (0.260)	−0.26
CV step length	0.16 ± 0.02	0.20 ± 0.05	U = 110, ***p* = 0.011 (0.033)**	**−0.48**
Average stride speed	0.50 ± 0.10	0.55 ± 0.10	U = 138, *p* = 0.068 (0.122)	−0.34
CV stride speed	0.17 ± 0.02	0.22 ± 0.07	U = 61, ***p* < 0.001 (0.009)**	**−0.71**
Average stride fluidity	7.8 ± 2.3	7.6 ± 1.4	U = 106, *p* = 0.929 (0.929)	−0.50
CV stride fluidity	0.25 ± 0.06	0.31 ± 0.08	U = 103, ***p* = 0.007 (0.032)**	**−0.51**

APD_LCIG_, patients with advanced Parkinson’s disease treated with L-Dopa/Carbidopa intestinal gel infusion; APD_L-Dopa_, patients with advanced Parkinson’s disease on best medical treatment with oral L-Dopa; CV, coefficient of variation. Bold values indicate statistically significant results.

**Figure 3:**
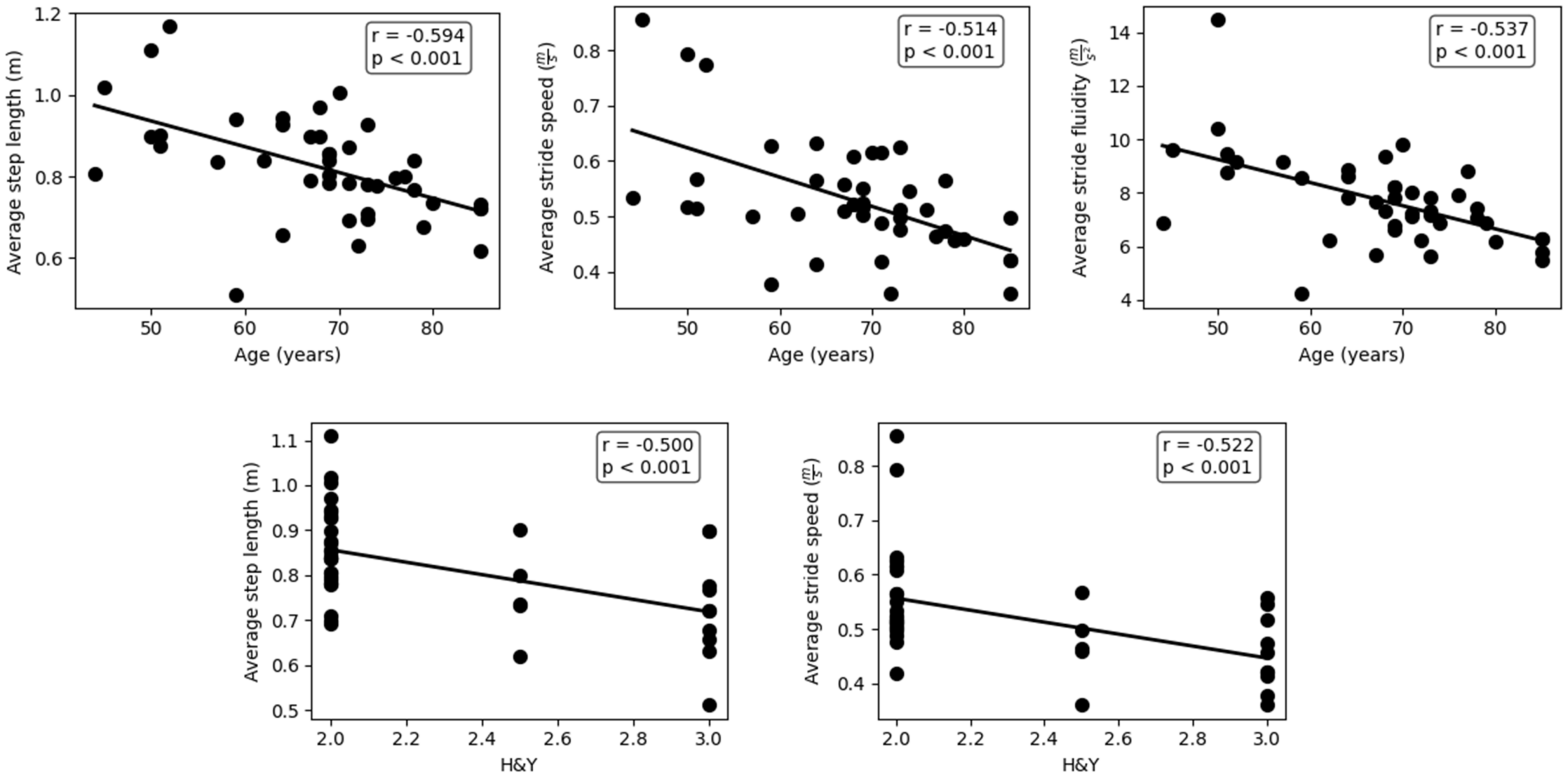
Clinical-behavioral correlations. Scatter plots illustrate the relationships between wearable-derived spatiotemporal gait parameters (including step length, stride speed, and stride fluidity) and clinical variables such as age and disease severity (Hoehn and Yahr stage – H&Y).

Among the spatiotemporal gait parameters, in the final analytical sample (*n =* 43), mean stride fluidity, step length, and stride speed exhibited moderate inverse correlations with age (r = −0.53 to −0.58, all *p* < 0.001), while step length and stride speed were also moderately and negatively associated with disease severity as reflected by H&Y stage (r = −0.50 and −0.52, respectively; both p < 0.001) (Figure 3). All these associations remained significant after correction for multiple comparisons (FDR, *p* < 0.05).

## 4 Discussion

In this observational, cross-sectional pilot study, long-term home monitoring of gait with a wearable sensor enabled an objective comparison of real-life mobility between two clinically matched cohorts of APD patients treated with either LCIG infusion or optimized oral dopaminergic therapy. Despite comparable disease duration and severity, APD_LCIG_ showed less severe motor fluctuations, as clinically shown by lower WOQ-19 scores and objectively indicated by reduced intra-day variability of spatiotemporal gait parameters, compared with APD_L-Dopa_. Overall, these findings suggest that LCIG therapy is associated with superior quality and stability of real-life movement compared with optimized oral dopaminergic treatment. From a clinical perspective, these results support the use of LCIG to reduce subjective motor fluctuations and improve the stability of motor performance in daily life.

To minimize potential confounding, we implemented strict inclusion criteria and several methodological precautions, with particular attention to clinical variables known to affect spatiotemporal gait parameters, including age, disease severity, orthopedic comorbidities, anxiety and mood disorders, and cognitive status. Although recruiting a clinically homogeneous cohort of patients with APD is inherently challenging, the two subgroups (APD_LCIG_ and APD_L-Dopa_) were carefully matched for all major demographic and clinical characteristics to ensure a reliable comparison. Participants also received clear, standardized instructions for sensor placement and use during home monitoring, with caregiver support encouraged when needed to further reduce the risk of handling errors.

A first finding of this study concerns the epidemiological distribution of the considered treatment modalities among the APD patients screened for eligibility. Approximately 25% of those meeting the inclusion criteria were receiving LCIG, whereas the remaining ∼75% were treated exclusively with best medical oral therapy. This proportion is largely consistent with observations from the literature (43–45). Although the exact percentage of APD patients treated with LCIG is not well established and varies substantially across health-care systems, real-world evidence consistently indicates that only a minority of APD patients receive any device-aided therapy, with estimates ranging from about 15% to over 40% across studies (43–45). In the present work we did not include APD patients treated with device-aided therapies other than LCIG infusion. Given the specialized expertise of the participating centers in movement disorders management, it is likely that the proportion of APD patients receiving a device-aided therapy would have been higher if patients treated with other device-aided options (such as deep brain stimulation or continuous subcutaneous apomorphine or L-Dopa infusion) had also been included. However, it is well established that, despite their proven value, device-aided therapies remain underused in real-world practice, largely because treatment decisions are often shaped by non-clinical factors rather than consistent guideline-based criteria (45, 46).

### 4.1 L-Dopa/Carbidopa intestinal gel infusion in advanced Parkinson’s disease

Our clinical and demographic matching of the two subgroups ensured that patients entered the comparison with largely similar baseline characteristics. As expected, APD_LCIG_ showed lower WOQ-19 scores than APD_L-Dopa_, indicating a reduced severity of both motor and non-motor fluctuations. This finding is fully consistent with evidence from clinical trials and real-world observational studies demonstrating that continuous dopaminergic delivery with LCIG substantially reduces OFF time, improves ON time, and significantly attenuates motor symptom variability (3, 47–50). This clinical advantage was objectively supported by long-term wearable sensor monitoring, which revealed markedly lower intra-day variability in key spatiotemporal gait parameters in our LCIG group. Differences in fluctuation-related gait dynamics would have otherwise remained difficult to capture without prolonged and ecologically valid monitoring. This gait pattern suggests that continuous dopaminergic delivery achieved through LCIG infusion not only reduces subjective fluctuation burden but also translates into more stable motor performance under real-life conditions. Clinically, this improved stability may be particularly relevant for activities of daily living, where consistent motor output, rather than peak performance, is required.

From a pathophysiological perspective, LCIG acts by smoothing intra-day motor fluctuations and limiting disabling dyskinesia, thereby leading patients to perceive an overall increase in ON time, an effect traditionally captured by clinical scales and patient diaries (51). This effect is consistent with the therapeutic rationale of LCIG, which aims to stabilize plasma L-Dopa levels and minimize the oscillations associated with intermittent oral dosing. Although not directly assessed in the present study, greater motor stability may plausibly translate into improved functional autonomy. This has potential implications for fall risk reduction and mobility-related disability. In line with this interpretation, increased stride-to-stride variability of spatiotemporal gait parameters is a well-established marker of impaired gait automaticity and instability, and has been consistently associated with reduced mobility and an increased risk of falls in both laboratory and real-life settings in PD (52–55). In general, although APD is often characterized by axial symptoms that are poorly responsive to dopaminergic therapy (56, 57), several spatiotemporal gait parameters remain sensitive to dopaminergic modulation (58, 59). Since changes in gait metrics have been shown to parallel the therapeutic responsiveness of appendicular motor functions (60), gait-based telemonitoring may offer a complementary approach for capturing global motor performance and informing treatment optimization in APD.

While we observed clearly increased gait variability in APD_L-Dopa_ compared with APD_LCIG_, the absolute values of spatiotemporal metrics as well as FOG occurrence were largely comparable between groups. At first glance, this might suggest that overall gait performance was similar. However, mean values of gait parameters are known to be relatively insensitive to short-term motor fluctuations and are strongly influenced by multiple individual and environmental interfering factors, which may mask between-group differences in APD (61–63). Individual and environmental factors may also contribute to substantial discrepancies in the absolute values of spatiotemporal gait parameters recorded in home-based versus supervised settings (64, 65). Indeed, mobility assessed in real-world, unsupervised conditions reflects complex interactions between physiological, behavioral, cognitive, and environmental influences, leading to potential discrepancies even when average values appear similar. Supervised or aggregated mean measures tend to capture a patient’s “best” performance rather than their typical day-to-day motor behavior, thereby reducing sensitivity to fluctuation-related impairment (66). This limitation is particularly relevant in APD, where motor complications are characterized by temporal instability rather than persistent deficits. Accordingly, similar mean values across groups do not exclude meaningful differences in the stability and consistency of motor output. This distinction has important clinical implications, as treatment-related benefits may remain undetected if only average gait performance or brief clinical assessments are considered. Moreover, it should be noted that, given the comparable disease severity between groups, distinct differences in the absolute values of spatiotemporal gait parameters and FOG occurrence would not be expected (67). In contrast, variability measures capture the moment-to-moment stability of motor control and therefore provide a more sensitive index of fluctuation-related impairment (61, 68, 69). From this perspective, the observed increase in gait variability in APD_L-Dopa_ likely reflects less stable motor control across the day, despite comparable average performance, in agreement with previous studies showing that variability-derived metrics better reflect real-world motor fluctuations than absolute spatiotemporal parameters alone (66, 70). Therefore, variability metrics may represent more sensitive and clinically meaningful digital measures for monitoring treatment response in APD. The significant correlations of spatiotemporal gait parameters with demographic and clinical features, including disease severity, further strengthen our observations. Indeed, these associations underscore the close interplay between overall clinical status and gait quality in free-living settings in PD (71, 72).

Further consideration concerns the observation of comparable time spent with dyskinesia in our APD_LCIG_ compared with APD_L-Dopa_. This result may appear in contrast with previous studies consistently reporting a reduction in troublesome dyskinesia burden following LCIG infusion (73–76). This apparent inconsistency may partially be related to differences in dyskinesia assessment methods across studies. Indeed, while the sensor quantifies the duration of dyskinetic activity, it does not provide information on dyskinesia severity. It is therefore plausible that APD_LCIG_ may exhibit more prolonged but mild dyskinesia, whereas APD_L-Dopa_ could experience more intense, short-term peak-dose dyskinesia associated with intermittent drug delivery. Such differences in phenomenology and severity would not be distinguished by the sensor but could nevertheless account for the observed similarity in dyskinesia duration. Accordingly, the comparable time spent with dyskinesia observed in APD_LCIG_ and APD_L-Dopa_ may reflect a shift from troublesome to non-troublesome dyskinesia in the APD_LCIG_ group, indicating a reduction in clinical impact despite similar overall duration. Supporting this interpretation, our APD_LCIG_ were clinically stable and did not report troublesome involuntary movements, yet they may have been unaware of subtle, low-amplitude trunk dyskinesia (77, 78). A possible impaired awareness of motor issues in APD further reinforces the need for quantitative, instrumented approaches such as wearable sensors, especially in advanced stages where clinical fluctuations become less reliably captured through patient self-report. Wearable sensors may be therefore particularly valuable in the context of APD, providing objective measures of dyskinesia and motor fluctuations that are not fully captured by clinical tools.

As an alternative hypothesis, despite an overall benefit at the group level, several reports have described a subset of patients who develop new or persistent dyskinesia after LCIG initiation, particularly diphasic or more complex patterns (74, 79–82). This paradoxical response has been attributed to an extremely narrow therapeutic window in some APD patients, maladaptive dopaminergic plasticity resulting from long-standing L-Dopa exposure, and challenges in titrating the optimal LCIG dose in individuals with heightened susceptibility (74). Given that the waist-mounted wearable used in our study captures dyskinesia involving both the trunk and lower limbs, it cannot be excluded that this phenomenon was particularly represented in our LCIG cohort. Lastly, an additional contributing factor may be the uneven sex distribution between groups, with a higher proportion of women in the APD_LCIG_ subgroup. Women with PD may indeed be more prone than men to developing L-Dopa-induced dyskinesia due to sex-related biological and pharmacokinetic factors (83–85).

### 4.2 Wearable sensors for the management of LCIG infusion

While our data do not directly address clinical decision-making or medication-titration, hypothetical clinical applications to suggest are promising. The clinical management of LCIG infusion presents several challenges, including patient selection, medication titration, and the longitudinal monitoring of treatment efficacy and safety, that may benefit from the integration of wearable sensor technology (86).

Regarding patient selection, in line with established clinical practice (87, 88), quantifying ≥2 h/day of OFF time and ≥1 h/day of troublesome dyskinesia is essential to identify APD patients inadequately controlled on optimized oral regimens who may be appropriate candidates for device-aided therapies such as LCIG infusion. These thresholds were originally derived from expert consensus and patient-reported motor diaries (88). High-resolution wearable monitoring may enable a data-driven recalibration of these cut-offs, better aligning them with real-world motor impairment and functional disability. Supporting this observation, our findings showed that long-term, home-based monitoring with a single wearable sensor could reliably quantify dynamic changes in patients’ motor status and the occurrence of dyskinesia, identifying distinct mobility patterns in patients treated with different therapeutic strategies. Wearable sensors used in free-living conditions may therefore enhance patient selection by providing objective, ecologically valid metrics that do not rely on patient or caregiver reporting and that may also have prognostic value in predicting treatment response, as previously demonstrated (28). This approach may help identify patients who are most likely to benefit from device-aided therapies, beyond conventional diary-based criteria.

With respect to medication titration, LCIG dose adjustment is not always conducted under prolonged in-hospital supervision. Indeed, in many centres, it is performed in outpatient settings through brief, repeated assessments (89, 90). Even during hospitalization, however, the immediate post–PEG-J initiation period does not fully reflect the patient’s home condition due to environmental and activity-level differences, as well as attentional influences (a Hawthorne-like behavioural effect) (91–93). Moreover, LCIG initiation often requires discontinuation of long half-life dopaminergic agents (e.g., dopamine agonists, enzyme inhibitors), which frequently necessitates additional LCIG adjustments after discharge, when continuous supervision is no longer possible (94). Prolonged home-based monitoring with wearable sensors can capture real-world motor performance, including intra-day variability, during this critical phase, thereby supporting a more accurate and individualized titration of LCIG. In clinical practice, this could translate into more efficient dose optimization and reduced need for repeated in-hospital assessments.

Finally, during chronic LCIG infusion, consistent clinical follow-up is required to maintain therapeutic efficacy and adapt infusion parameters to evolving clinical needs, including progression of motor fluctuations or emergence of dyskinesia (94). Wearable sensors provide objective, continuous information on motor status that can reveal subtle deterioration or suboptimal treatment control, allowing clinicians to modify infusion settings timely and sustain long-term treatment effectiveness.

This study has some limitations to be acknowledged. First, despite multi-day monitoring, the cross-sectional analytical design prevents causal inference, limiting the interpretation of between-group differences. The observed associations should be therefore interpreted as descriptive rather than indicative of treatment effects, as potential confounding factors and selection biases cannot be fully excluded. In particular, the lack of longitudinal data prevents within-patient comparisons over time, which would allow for a more reliable evaluation of treatment effects while minimizing the influence of inter-individual variability. Longitudinal and randomized studies are warranted to confirm our findings and establish causality. Second, the relatively small sample size, particularly in the APD_LCIG_ group, may reduce statistical power and limit the generalizability of the findings. In addition, the sex imbalance between subgroups may have contributed to variability and potential residual confounding. Therefore, the results should be interpreted with caution and confirmed in larger cohorts. Third, despite careful matching for key demographic and clinical variables and for overall physical activity (estimated by daily step count), residual heterogeneity cannot be excluded. Indeed, confounding may derive from unmeasured or incompletely captured factors, such as differences in motor fluctuation patterns, non-motor burden, or environmental influences on patients’ daily activity. Fourth, the wearable sensor quantifies dyskinesia duration but cannot distinguish its phenomenology or severity, which may vary across patients and treatment groups. Overall, larger studies with extended, repeated monitoring will be required to validate and generalize our findings.

## 5 Conclusion

This cross-sectional, observational pilot study provides preliminary objective and quantitative evidence suggesting that LCIG is associated with a more stable gait pattern than optimized oral dopaminergic therapy. Although causal interpretations cannot be established, this effect likely reflects more consistent motor control throughout the day due to continuous dopaminergic delivery, potentially resulting in overall improved physical activity. By capturing fluctuation-related motor impairment through gait variability metrics, wearable sensor technology may represent a valuable tool to enhance the clinical management of LCIG, supporting patient selection, medication titration, and the longitudinal monitoring of treatment efficacy and safety.

Future research should assess whether incorporating wearable sensors into the main phases of LCIG management, including patient selection, medication titration and the longitudinal monitoring of treatment efficacy and safety, results in meaningful clinical advantages. Extending this methodological approach to other device-aided therapies, such as alternative infusion systems (e.g., subcutaneous L-Dopa or apomorphine) or deep brain stimulation, may further clarify the value of continuous real-life motor monitoring in APD. Lastly, the integration of sensor-derived metrics into adaptive or closed-loop therapeutic frameworks could enable more precise and personalized treatment strategies for patients with APD.

## Data Availability

The raw data supporting the conclusions of this article will be made available by the corresponding author upon reasonable request.

## References

[1] Aslam S Manfredsson F Stokes A Shill H. «advanced» Parkinson’s disease: a review. Parkinsonism Relat Disord. (2024) 123:106065. doi: 10.1016/j.parkreldis.2024.106065, 38418318

[2] Tsunemi T Oyama G Saiki S Hatano T Fukae J Shimo Y . Intrajejunal infusion of levodopa/carbidopa for advanced Parkinson’s disease: a systematic review. Mov Disord. (2021) 36:1759–71. doi: 10.1002/mds.28595, 33899262 PMC9290931

[3] Antonini A Poewe W Chaudhuri KR Jech R Pickut B Pirtošek Z . Levodopa-carbidopa intestinal gel in advanced Parkinson’s: final results of the GLORIA registry. Parkinsonism Relat Disord. (2017) 45:13–20. doi: 10.1016/j.parkreldis.2017.09.018, 29037498

[4] Fernandez HH Boyd JT Fung VSC Lew MF Rodriguez RL Slevin JT . Long-term safety and efficacy of levodopa-carbidopa intestinal gel in advanced Parkinson’s disease. Mov Disord. (2018) 33:928–36. doi: 10.1002/mds.27338, 29570853

[5] Lopiano L Modugno N Marano P Sensi M Meco G Solla P . Motor and non-motor outcomes in patients with advanced Parkinson’s disease treated with levodopa/carbidopa intestinal gel: final results of the GREENFIELD observational study. J Neurol. (2019) 266:2164–76. doi: 10.1007/s00415-019-09337-6, 31134377 PMC6687881

[6] Murata M Mihara M Hasegawa K Jeon B Tsai CH Nishikawa N . Safety and efficacy of levodopa-carbidopa intestinal gel: results from an open-label extension study in Japanese, Korean and Taiwanese patients with advanced Parkinson’s disease. Ther Adv Neurol Disord. (2018) 11:1756286418759315. doi: 10.1177/1756286418759315, 29511383 PMC5833238

[7] Wang L Li J Chen J. Levodopa-carbidopa intestinal gel in Parkinson’s disease: a systematic review and Meta-analysis. Front Neurol. (2018) 9:620. doi: 10.3389/fneur.2018.00620, 30104997 PMC6077236

[8] Krüger R Lingor P Doskas T Henselmans JML Danielsen EH de Fabregues O . An observational study of the effect of levodopa-carbidopa intestinal gel on activities of daily living and quality of life in advanced Parkinson’s disease patients. Adv Ther. (2017) 34:1741–52. doi: 10.1007/s12325-017-0571-2, 28631218 PMC5504221

[9] Garrì F Russo FP Carrer T Weis L Pistonesi F Mainardi M . Long-term safety, discontinuation and mortality in an Italian cohort with advanced Parkinson’s disease on levodopa/carbidopa intestinal gel infusion. J Neurol. (2022) 269:5606–14. doi: 10.1007/s00415-022-11269-7, 35876875 PMC9309989

[10] Stone AA Shiffman S Schwartz JE Broderick JE Hufford MR. Patient compliance with paper and electronic diaries. Control Clin Trials. (2003) 24:182–99. doi: 10.1016/s0197-2456(02)00320-3, 12689739

[11] Maetzler W Domingos J Srulijes K Ferreira JJ Bloem BR. Quantitative wearable sensors for objective assessment of Parkinson’s disease. Mov Disord. (2013) 28:1628–37. doi: 10.1002/mds.25628, 24030855

[12] Ossig C Antonini A Buhmann C Classen J Csoti I Falkenburger B . Wearable sensor-based objective assessment of motor symptoms in Parkinson’s disease. J Neural Transm. (2016) 123:57–64. doi: 10.1007/s00702-015-1439-8, 26253901

[13] Moreau C Rouaud T Grabli D Benatru I Remy P Marques AR . Overview on wearable sensors for the management of Parkinson’s disease. NPJ Parkinsons Dis. (2023) 9:153. doi: 10.1038/s41531-023-00585-y, 37919332 PMC10622581

[14] Correno MB Hansen C Carlin T Vuillerme N. Objective measurement of walking activity using wearable technologies in people with Parkinson disease: a systematic review. Sensors. (2022) 22:4551. doi: 10.3390/s22124551, 35746329 PMC9229799

[15] Adams JL Dinesh K Snyder CW Xiong M Tarolli CG Sharma S . A real-world study of wearable sensors in Parkinson’s disease. NPJ Parkinsons Dis. (2021) 7:106. doi: 10.1038/s41531-021-00248-w, 34845224 PMC8629990

[16] Zampogna A Borzì L Rinaldi D Artusi CA Imbalzano G Patera M . Unveiling the unpredictable in Parkinson’s disease: sensor-based monitoring of dyskinesias and freezing of gait in daily life. Bioengineering. (2024) 11:440. doi: 10.3390/bioengineering11050440, 38790307 PMC11117481

[17] Wu X Ma L Wei P Shan Y Chan P Wang K . Wearable sensor devices can automatically identify the ON-OFF status of patients with Parkinson’s disease through an interpretable machine learning model. Front Neurol. (2024) 15:1387477. doi: 10.3389/fneur.2024.1387477, 38751881 PMC11094303

[18] Zampogna A Mileti I Palermo E Celletti C Paoloni M Manoni A . Fifteen years of wireless sensors for balance assessment in neurological disorders. Sensors. (2020) 20:3247. doi: 10.3390/s20113247, 32517315 PMC7308812

[19] Suppa A Kita A Leodori G Zampogna A Nicolini E Lorenzi P . L-DOPA and freezing of gait in Parkinson’s disease: objective assessment through a wearable wireless system. Front Neurol. (2017) 8:406. doi: 10.3389/fneur.2017.00406, 28855889 PMC5557738

[20] Zampogna A Mileti I Martelli F Paoloni M Del Prete Z Palermo E . Early balance impairment in Parkinson’s disease: evidence from robot-assisted axial rotations. Clin Neurophysiol. (2021) 132:2422–30. doi: 10.1016/j.clinph.2021.06.023, 34454269

[21] Botros A Schütz N Camenzind M Urwyler P Bolliger D Vanbellingen T . Long-term home-monitoring sensor technology in patients with Parkinson’s disease-acceptance and adherence. Sensors. (2019) 19:5169. doi: 10.3390/s19235169, 31779108 PMC6928790

[22] Kangarloo T Latzman RD Adams JL Dorsey R Kostrzebski M Severson J . Acceptability of digital health technologies in early Parkinson’s disease: lessons from WATCH-PD. Front Digit Health. (2024) 6:1435693. doi: 10.3389/fdgth.2024.1435693, 39253055 PMC11381495

[23] Virbel-Fleischman C Mousin F Liu S Hardy S Corvol JC Benatru I . Symptoms assessment and decision to treat patients with advanced Parkinson’s disease based on wearables data. NPJ Parkinsons Dis. (2023) 9:45. doi: 10.1038/s41531-023-00489-x, 36973302 PMC10042860

[24] Pahwa R Isaacson SH Torres-Russotto D Nahab FB Lynch PM Kotschet KE. Role of the personal KinetiGraph in the routine clinical assessment of Parkinson’s disease: recommendations from an expert panel. Expert Rev Neurother. (2018) 18:669–80. doi: 10.1080/14737175.2018.1503948 30032695

[25] Johansson D Ericsson A Johansson A Medvedev A Nyholm D Ohlsson F . Individualization of levodopa treatment using a microtablet dispenser and ambulatory accelerometry. CNS Neurosci Ther. (2018) 24:439–47. doi: 10.1111/cns.12807, 29652438 PMC6490091

[26] Thomas I Alam M Bergquist F Johansson D Memedi M Nyholm D . Sensor-based algorithmic dosing suggestions for oral administration of levodopa/carbidopa microtablets for Parkinson’s disease: a first experience. J Neurol. (2019) 266:651–8. doi: 10.1007/s00415-019-09183-6, 30659356 PMC6394802

[27] Heldman DA Giuffrida JP Cubo E. Wearable sensors for advanced therapy referral in Parkinson’s disease. J Parkinsons Dis. (2016) 6:631–8. doi: 10.3233/JPD-160830, 27392872

[28] Kilinçalp G Sjöström AC Eriksson B Holmberg B Constantinescu R Bergquist F. Predictive value of ambulatory objective movement measurement for outcomes of levodopa/carbidopa intestinal gel infusion. J Pers Med. (2022) 12:27. doi: 10.3390/jpm12010027, 35055343 PMC8781512

[29] Noui Y Silverdale MA Evans J Partington-Smith L Kobylecki C. Parkinson’s kinetigraph in the selection of levodopa-carbidopa intestinal gel for motor fluctuations refractory to deep brain stimulation. J Mov Disord. (2021) 14:239–41. doi: 10.14802/jmd.20090, 33706473 PMC8490188

[30] Evers LJ Raykov YP Krijthe JH de Silva Lima AL Badawy R Claes K . Real-life gait performance as a digital biomarker for motor fluctuations: the Parkinson@home validation study. J Med Internet Res. (2020) 22:e19068. doi: 10.2196/19068, 33034562 PMC7584982

[31] Pfister FMJ Um TT Pichler DC Goschenhofer J Abedinpour K Lang M . High-resolution motor state detection in Parkinson’s disease using convolutional neural networks. Sci Rep. (2020) 10:5860. doi: 10.1038/s41598-020-61789-3, 32246097 PMC7125162

[32] Imbalzano G Artusi CA Ledda C Montanaro E Romagnolo A Rizzone MG . Effects of continuous dopaminergic stimulation on Parkinson’s disease gait: a longitudinal prospective study with levodopa intestinal gel infusion. J Parkinsons Dis. (2024) 14:843–53. doi: 10.3233/JPD-240003, 38728203 PMC11191481

[33] Postuma RB Berg D Stern M Poewe W Olanow CW Oertel W . MDS clinical diagnostic criteria for Parkinson’s disease. Mov Disord. (2015) 30:1591–601. doi: 10.1002/mds.26424, 26474316

[34] Moes HR Henriksen T Sławek J Phokaewvarangkul O Buskens E van Laar T. Tools and criteria to select patients with advanced Parkinson’s disease for device-aided therapies: a narrative review. J Neural Transm. (2023) 130:37500937:1359–77. doi: 10.1007/s00702-023-02656-z PMC10645650 37500937

[35] Jost ST Kaldenbach MA Antonini A Martinez-Martin P Timmermann L Odin P . Levodopa dose equivalency in Parkinson’s disease: updated systematic review and proposals. Mov Disord Off J Mov Disord Soc Luglio. (2023) 38:1236–52. doi: 10.1002/mds.29410, 37147135

[36] Santos García D López Ariztegui N Cubo E Vinagre Aragón A García-Ramos R Borrué C . Clinical utility of a personalized and long-term monitoring device for Parkinson’s disease in a real clinical practice setting: an expert opinion survey on STAT-ON™. Neurologia. (2023) 38:326–33. doi: 10.1016/j.nrl.2020.10.013, 37263727

[37] Rodríguez-Martín D Cabestany J Pérez-López C Pie M Calvet J Samà A . A new paradigm in Parkinson’s disease evaluation with wearable medical devices: a review of STAT-ONTM. Front Neurol. (2022) 13:912343. doi: 10.3389/fneur.2022.912343, 35720090 PMC9202426

[38] Bianchini E Galli S Alborghetti M De Carolis L Zampogna A Hansen C . Four days are enough to provide a reliable daily step count in mild to moderate Parkinson’s disease through a commercial smartwatch. Sensors. (2023) 23:18971. doi: 10.3390/s23218971, 37960670 PMC10649244

[39] Sayeed T Samà A Català A Rodríguez-Molinero A Cabestany J. Adapted step length estimators for patients with Parkinson’s disease using a lateral belt worn accelerometer. Technol Health Care Off J Eur Soc Eng Med. (2015) 23:179–94. doi: 10.3233/THC-140882 25468759

[40] Samà A Pérez-Lopez C Romagosa J Rodríguez-Martín D Català A Cabestany J . Dyskinesia and motor state detection in Parkinson’s disease patients with a single movement sensor. Annu Int Conf IEEE Eng Med Biol Soc IEEE Eng Med Biol Soc Annu Int Conf. (2012) 2012:1194–7. doi: 10.1109/EMBC.2012.6346150, 23366111

[41] Samà A Pérez-López C Rodríguez-Martín D Català A Moreno-Aróstegui JM Cabestany J . Estimating bradykinesia severity in Parkinson’s disease by analysing gait through a waist-worn sensor. Comput Biol Med. (2017) 84:114–23. doi: 10.1016/j.compbiomed.2017.03.020, 28351715

[42] Rodríguez-Martín D Samà A Pérez-López C Català A Moreno Arostegui JM Cabestany J . Home detection of freezing of gait using support vector machines through a single waist-worn triaxial accelerometer. PLoS One. (2017) 12:e0171764. doi: 10.1371/journal.pone.0171764, 28199357 PMC5310916

[43] Santos-García D González-Ortega G Sánchez-Alonso P Planas-Ballvé A García-Ramos R Cabo I . Device-aided therapies (DATs) in Parkinson’s disease (PD). The DATs-PD GETM Spanish registry protocol study. PLoS One. (2025) 20:e0316052. doi: 10.1371/journal.pone.0316052 40163422 PMC11957350

[44] Fasano A Fung VSC Seppi K Pirtosek Z Takáts A Alobaidi A . Intercountry comparisons of advanced Parkinson’s disease symptoms and management: analysis from the OBSERVE-PD observational study. Acta Neurol Scand. (2022) 146:167–76. doi: 10.1111/ane.13648, 35607843 PMC9541702

[45] Auffret M Weiss D Stocchi F Vérin M Jost WH. Access to device-aided therapies in advanced Parkinson’s disease: navigating clinician biases, patient preference, and prognostic uncertainty. J Neural Transm. (2023) 130:1411–32. doi: 10.1007/s00702-023-02668-9, 37436446 PMC10645670

[46] Lökk J. Lack of information and access to advanced treatment for Parkinson’s disease patients. J Multidiscip Healthc. (2011) 4:433–9. doi: 10.2147/JMDH.S27180, 22247618 PMC3256003

[47] Fernandez HH Standaert DG Hauser RA Lang AE Fung VSC Klostermann F . Levodopa-carbidopa intestinal gel in advanced Parkinson’s disease: final 12-month, open-label results. Mov Disord Off J Mov Disord Soc. (2015) 30:500–9. doi: 10.1002/mds.26123, 25545465 PMC4674978

[48] Chaudhuri KR Antonini A Pahwa R Odin P Titova N Thakkar S . Effects of levodopa-carbidopa intestinal gel on dyskinesia and non-motor symptoms including sleep: results from a meta-analysis with 24-month follow-up. J Parkinsons Dis. (2021) 12:2071–83. doi: 10.3233/JPD-223295, 35964203 PMC9661331

[49] Olanow CW Kieburtz K Odin P Espay AJ Standaert DG Fernandez HH . Continuous intrajejunal infusion of levodopa-carbidopa intestinal gel for patients with advanced Parkinson’s disease: a randomised, controlled, double-blind, double-dummy study. Lancet Neurol. (2014) 13:141–9. doi: 10.1016/S1474-4422(13)70293-X, 24361112 PMC4643396

[50] Othman AA Chatamra K Mohamed MEF Dutta S Benesh J Yanagawa M . Jejunal infusion of levodopa-carbidopa intestinal gel versus oral administration of levodopa-carbidopa tablets in Japanese subjects with advanced Parkinson’s disease: pharmacokinetics and pilot efficacy and safety. Clin Pharmacokinet. (2015) 54:975–84. doi: 10.1007/s40262-015-0265-3, 25875940 PMC4559582

[51] Antonini A Odin P Pahwa R Aldred J Alobaidi A Jalundhwala YJ . The long-term impact of levodopa/carbidopa intestinal gel on ‘off’-time in patients with advanced Parkinson’s disease: a systematic review. Adv Ther. (2021) 38:2854–90. doi: 10.1007/s12325-021-01747-1, 34018146 PMC8189983

[52] Hausdorff JM Cudkowicz ME Firtion R Wei JY Goldberger AL. Gait variability and basal ganglia disorders: stride-to-stride variations of gait cycle timing in Parkinson’s disease and Huntington’s disease. Mov Disord Off J Mov Disord Soc. (1998) 13:428–37. doi: 10.1002/mds.870130310, 9613733

[53] Kwon KY You J Kim RO Lee EJ Lee J Kim I . Association between baseline gait parameters and future fall risk in patients with de novo Parkinson’s disease: forward versus backward gait. J Clin Neurol. (2024) 20:201–7. doi: 10.3988/jcn.2022.0299, 38171499 PMC10921052

[54] Ellis RJ Ng YS Zhu S Tan DM Anderson B Schlaug G . A validated smartphone-based assessment of gait and gait variability in Parkinson’s disease. PLoS One. (2015) 10:e0141694. doi: 10.1371/journal.pone.0141694, 26517720 PMC4627774

[55] Shah VV Jagodinsky A McNames J Carlson-Kuhta P Nutt JG El-Gohary M . Gait and turning characteristics from daily life increase ability to predict future falls in people with Parkinson’s disease. Front Neurol. (2023) 14:1096401. doi: 10.3389/fneur.2023.1096401, 36937534 PMC10015637

[56] Zampogna A Cavallieri F Bove F Suppa A Castrioto A Meoni S . Axial impairment and falls in Parkinson’s disease: 15 years of subthalamic deep brain stimulation. NPJ Parkinsons Dis. (2022) 8:121. doi: 10.1038/s41531-022-00383-y, 36153351 PMC9509398

[57] Fasano A Aquino CC Krauss JK Honey CR Bloem BR. Axial disability and deep brain stimulation in patients with Parkinson disease. Nat Rev Neurol. (2015) 11:98–110. doi: 10.1038/nrneurol.2014.252, 25582445

[58] Curtze C Nutt JG Carlson-Kuhta P Mancini M Horak FB. Levodopa is a double-edged sword for balance and gait in people with Parkinson’s disease. Mov Disord. (2015) 30:1361–70. doi: 10.1002/mds.26269, 26095928 PMC4755510

[59] Imbalzano G Rinaldi D Calandra-Buonaura G Contin M Amato F Giannini G . How resistant are levodopa-resistant axial symptoms? Response of freezing, posture, and voice to increasing levodopa intestinal infusion rates in Parkinson disease. Eur J Neurol. (2023) 30:96–106. doi: 10.1111/ene.15558, 36093563 PMC10092343

[60] Zampogna A Pietrosanti L Saggio G Patera M Falletti M Bellia V . L-Dopa comparably improves gait and limb movements in Parkinson’s disease: a wearable sensor analysis. Biomedicine. (2025) 13:2727. doi: 10.3390/biomedicines13112727, 41301819 PMC12650135

[61] Bryant MS Rintala DH Hou JG Charness AL Fernandez AL Collins RL . Gait variability in Parkinson’s disease: influence of walking speed and dopaminergic treatment. Neurol Res. (2011) 33:959–64. doi: 10.1179/1743132811Y.0000000044, 22080998 PMC5361771

[62] Del Din S Godfrey A Galna B Lord S Rochester L. Free-living gait characteristics in ageing and Parkinson’s disease: impact of environment and ambulatory bout length. J Neuroeng Rehabil. (2016) 13:46. doi: 10.1186/s12984-016-0154-5, 27175731 PMC4866360

[63] Hausdorff JM. Gait variability: methods, modeling and meaning. J Neuroeng Rehabil. (2005) 2:19. doi: 10.1186/1743-0003-2-19, 16033650 PMC1185560

[64] Paulhus L Bernard K Bourque C Guilmette S Laquerre MÊ Laramée ELÈ . An updated synthesis of environmental factors influencing mobility and social participation in older adults: results from a scoping review. BMC Public Health. (2025) 26:109. doi: 10.1186/s12889-025-25849-5, 41345601 PMC12781921

[65] Roussos G Herrero TR Hill DL Dowling AV L T M Müller M Evers LJW . Identifying and characterising sources of variability in digital outcome measures in Parkinson’s disease. NPJ Digit Med. (2022) 5:93. doi: 10.1038/s41746-022-00643-4, 35840653 PMC9284971

[66] Warmerdam E Hausdorff JM Atrsaei A Zhou Y Mirelman A Aminian K . Long-term unsupervised mobility assessment in movement disorders. Lancet Neurol. (2020) 19:462–70. doi: 10.1016/S1474-4422(19)30397-7, 32059811

[67] Fasano A Mancini M. Wearable-based mobility monitoring: the long road ahead. Lancet Neurol. (2020) 19:378–9. doi: 10.1016/S1474-4422(20)30033-8 32059810

[68] Godi M Arcolin I Giardini M Corna S Schieppati M. A pathophysiological model of gait captures the details of the impairment of pace/rhythm, variability and asymmetry in parkinsonian patients at distinct stages of the disease. Sci Rep. (2021) 11:21143. doi: 10.1038/s41598-021-00543-9, 34707168 PMC8551236

[69] König N Singh NB Baumann CR Taylor WR. Can gait signatures provide quantitative measures for aiding clinical decision-making? A systematic meta-analysis of gait variability behavior in patients with Parkinson’s disease. Front Hum Neurosci. (2016) 10:319. doi: 10.3389/fnhum.2016.00319, 27445759 PMC4927578

[70] Din SD Godfrey A Mazzà C Lord S Rochester L. Free‐living monitoring of Parkinson's disease: Lessons from the field. Movement Disorders. 31:1293–313. doi: 10.1002/mds.26718 27452964

[71] Zhang X Jin Y Wang M Ji C Chen Z Fan W . The impact of anxiety on gait impairments in Parkinson’s disease: insights from sensor-based gait analysis. J Neuroeng Rehabil. (2024) 21:68. doi: 10.1186/s12984-024-01364-3, 38689288 PMC11059709

[72] Mirelman A Bonato P Camicioli R Ellis TD Giladi N Hamilton JL . Gait impairments in Parkinson’s disease. Lancet Neurol. (2019) 18:697–708. doi: 10.1016/S1474-4422(19)30044-4, 30975519

[73] Freire-Alvarez E Kurča E Lopez Manzanares L Pekkonen E Spanaki C Vanni P . Levodopa-carbidopa intestinal gel reduces dyskinesia in Parkinson’s disease in a randomized trial. Mov Disord. (2021) 36:2615–23. doi: 10.1002/mds.28703, 34236101 PMC9292774

[74] Fabbri M Zibetti M Calandra-Buonaura G Contin M Sambati L Mohamed S . Levodopa/carbidopa intestinal gel long-term outcome in Parkinson’s disease: focus on dyskinesia. Mov Disord Clin Pract. (2020) 7:930–9. doi: 10.1002/mdc3.13068, 33163564 PMC7604637

[75] Fasano A Spanaki C Gurevich T Jech R Svenningsson P Szász J . Levodopa-carbidopa intestinal gel improves dyskinesia in Parkinson’s disease: post hoc analysis from the COSMOS study. Mov Disord Clin Pract. 13:70222. doi: 10.1002/mdc3.70222, 40814840 PMC12839488

[76] Lopiano L Modugno N Marano P Sensi M Meco G Cannas A . Motor outcomes in patients with advanced Parkinson’s disease treated with levodopa/carbidopa intestinal gel in Italy: an interim analysis from the GREENFIELD observational study. Neurol Sci. (2016) 37:1785–92. doi: 10.1007/s10072-016-2664-0, 27421834 PMC5065887

[77] Amanzio M Monteverdi S Giordano A Soliveri P Filippi P Geminiani G. Impaired awareness of movement disorders in Parkinson’s disease. Brain Cogn. (2010) 72:337–46. doi: 10.1016/j.bandc.2009.10.011 19914762

[78] Amanzio M Palermo S Zibetti M Leotta D Rosato R Geminiani G . Self-unawareness of levodopa induced dyskinesias in patients with Parkinson’s disease. Brain Cogn. (2014) 90:135. doi: 10.1016/j.bandc.2014.06.014 25058494

[79] Meloni M Solla P Mascia MM Marrosu F Cannas A. Diphasic dyskinesias during levodopa-carbidopa intestinal gel (LCIG) infusion in Parkinson’s disease. Parkinsonism Relat Disord. (2017) 37:92–6. doi: 10.1016/j.parkreldis.2016.12.030, 28063683

[80] Marano M Naranian T di Biase L Di Santo A Poon YY Arca R . Complex dyskinesias in Parkinson patients on levodopa/carbidopa intestinal gel. Parkinsonism Relat Disord. (2019) 69:140–6. doi: 10.1016/j.parkreldis.2019.11.008, 31759188

[81] Buongiorno M Antonelli F Cámara A Puente V de Fabregues-Nebot O Hernandez-Vara J . Long-term response to continuous duodenal infusion of levodopa/carbidopa gel in patients with advanced Parkinson disease: the Barcelona registry. Parkinsonism Relat Disord. (2015) 21:871–6. doi: 10.1016/j.parkreldis.2015.05.014, 26003410

[82] Szász JA Constantin VA Orbán-Kis K Bancu LA Ciorba M Mihály I . Management challenges of severe, complex dyskinesia. Data from a large cohort of patients treated with levodopa-carbidopa intestinal gel for advanced Parkinson’s disease. Brain Sci. (2021) 11:826. doi: 10.3390/brainsci11070826, 34206596 PMC8301838

[83] Pellecchia MT Picillo M Russillo MC Amboni M Arabia G Avanzino L . Gender is the main predictor of wearing-off and dyskinesia in levodopa-naïve patients with Parkinson’s disease. Mov Disord Clin Pract. (2025) 12:1774–83. doi: 10.1002/mdc3.70143, 40439072 PMC12625146

[84] Cattaneo C Pagonabarraga J. Sex differences in Parkinson’s disease: a narrative review. Neurol Ther. (2025) 14:57–70. doi: 10.1007/s40120-024-00687-6, 39630386 PMC11762054

[85] Jung JH Chung SJ Yoo HS Lee YH Baik K Ye BS . Sex-specific association of urate and levodopa-induced dyskinesia in Parkinson’s disease. Eur J Neurol. (2020) 27:1948–56. doi: 10.1111/ene.14337, 32441832

[86] Rus T Premzl M Križnar NZ Kramberger MG Rajnar R Ocepek L . Adverse effects of levodopa/carbidopa intrajejunal gel treatment: a single-center long-term follow-up study. Acta Neurol Scand. (2022) 146:537–44. doi: 10.1111/ane.13675, 35903042 PMC9796727

[87] Antonini A Odin P Schmidt P Cubillos F Standaert DG Henriksen T . Validation and clinical value of the MANAGE-PD tool: a clinician-reported tool to identify Parkinson’s disease patients inadequately controlled on oral medications. Parkinsonism Relat Disord. (2021) 92:59–66. doi: 10.1016/j.parkreldis.2021.10.009, 34695657

[88] Santos-García D de Deus Fonticoba T Suárez Castro E Aneiros Díaz A McAfee D. 5-2-1 criteria: a simple screening tool for identifying advanced PD patients who need an optimization of Parkinson’s treatment. Parkinson's Dis. (2020) 2020:7537924. doi: 10.1155/2020/7537924, 32269748 PMC7128051

[89] Thomsen TH Nielsen NS Isenberg AL Møller MH Clausen JB Schack Frederiksen IM . Home-based titration with duodenal infusion of levodopa-carbidopa intestinal gel in people with Parkinson’s disease: an observational feasibility study. Parkinson's Dis. (2024) 2024:5522824. doi: 10.1155/2024/5522824, 38623494 PMC11018374

[90] Amjad F Bhatti D Davis TL Oguh O Pahwa R Kukreja P . Current practices for outpatient initiation of levodopa-carbidopa intestinal gel for Management of Advanced Parkinson’s disease in the United States. Adv Ther. (2019) 36:2233–46. doi: 10.1007/s12325-019-01014-4, 31278691 PMC6822848

[91] Atrsaei A Corrà MF Dadashi F Vila-Chã N Maia L Mariani B . Gait speed in clinical and daily living assessments in Parkinson’s disease patients: performance versus capacity. NPJ Parkinsons Dis. (2021) 7:24. doi: 10.1038/s41531-021-00171-0, 33674597 PMC7935857

[92] Toosizadeh N Mohler J Lei H Parvaneh S Sherman S Najafi B. Motor performance assessment in Parkinson’s disease: association between objective in-clinic, objective in-home, and subjective/semi-objective measures. PLoS One. (2015) 10:e0124763. doi: 10.1371/journal.pone.0124763, 25909898 PMC4409065

[93] Nishi Y Fujii S Ikuno K Terasawa Y Morioka S. Adjustability of gait speed in clinics and free-living environments for people with Parkinson’s disease. J Mov Disord. (2024) 17:416–24. doi: 10.14802/jmd.24167, 39313236 PMC11540547

[94] Burack M Aldred J Zadikoff C Vanagunas A Klos K Bilir B . Implementing levodopa-carbidopa intestinal gel for Parkinson disease: Insights from US practitioners. Mov Disord Clin Pract. (2018) 5:383–93. doi: 10.1002/mdc3.12630, 30363427 PMC6174493

